# A gene-based survival score for lung adenocarcinoma by multiple transcriptional datasets analysis

**DOI:** 10.1186/s12885-020-07473-1

**Published:** 2020-10-31

**Authors:** Yanlu Xiong, Jie Lei, Jinbo Zhao, Qiang Lu, Yangbo Feng, Tianyun Qiao, Shaowei Xin, Yong Han, Tao Jiang

**Affiliations:** 1 Department of Thoracic Surgery, Tangdu Hospital, Fourth Military Medical University, 569 Xinsi Road, Baqiao District, Xi’an City, 710038 Shaanxi Province China; 2 Department of Thoracic Surgery, Air Force Medical Center, PLA, 30 Fucheng Road, Haidian District, Beijing, 100142 China

**Keywords:** Lung adenocarcinoma, Transcriptome, Survival, Prediction, Risk

## Abstract

**Background:**

Lung adenocarcinoma (LUAD) remains a crucial factor endangering human health. Gene-based clinical predictions could be of great help for cancer intervention strategies. Here, we tried to build a gene-based survival score (SS) for LUAD via analyzing multiple transcriptional datasets.

**Methods:**

We first acquired differentially expressed genes between tumors and normal tissues from intersections of four LUAD datasets. Next, survival-related genes were preliminarily unscrambled by univariate Cox regression and further filtrated by LASSO regression. Then, we applied PCA to establish a comprehensive SS based on survival-related genes. Subsequently, we applied four independent LUAD datasets to evaluate prognostic prediction of SS. Moreover, we explored associations between SS and clinicopathological features. Furthermore, we assessed independent predictive value of SS by multivariate Cox analysis and then built prognostic models based on clinical stage and SS. Finally, we performed pathway enrichments analysis and investigated immune checkpoints expression underlying SS in four datasets.

**Results:**

We established a 13 gene-based SS, which could precisely predict OS and PFS of LUAD. Close relations were elicited between SS and canonical malignant indictors. Furthermore, SS could serve as an independent risk factor for OS and PFS. Besides, the predictive efficacies of prognostic models were also reasonable (C-indexes: OS, 0.7; PFS, 0.7). Finally, we demonstrated enhanced cell proliferation and immune escape might account for high clinical risk of SS.

**Conclusions:**

We built a 13 gene-based SS for prognostic prediction of LUAD, which exhibited wide applicability and could contribute to LUAD management.

## Background

Lung cancer remains intractable but imperative to cope with for the highest morbidity and mortality among cancers [1]. A principle subtype of lung cancers is lung adenocarcinoma (LUAD), whose investigation means a great deal to us [2–4]. Advance in cancer biology demonstrated cancer could be regarded as a disorder caused mainly by aberrant genes, while some core ones even drive carcinogenesis [5, 6]. That is to say, genes are undoubtedly valuable targets for cancer management.

In fact, remarkable achievements in clinical practice have proved powerful effect of genes on clinical oncology especially for LUAD [7, 8]. First take chemotherapy for example. Many widely applied chemotherapeutic agents are aimed at critical genes in biological processes like cell proliferation and metabolism [9, 10]. Besides, targeted therapy based on driver gene, such as epidermal growth factor receptor (EGFR), has significantly improved the prognosis of patients with specific genetic background [11, 12]. Moreover, immunotherapy targeted at immune-checkpoint genes has achieved revolutionary progress for LUAD patients, especially for who have no targetable driver mutation till now [4, 7, 13].

Moreover, clinical predictions based on gene signatures also contribute much to handling cancer [14–16]. For example, some canonical biomarkers are references for distinguishing specific cancer from normal counterparts and other histologic subtypes [17, 18]. Besides, other gene-based clinical prediction like prognostic prediction has emerged as a hot spot for the relative convenience to obtain and the great significance for treatment [15]. Tremendous advance in omics and mature application of statistical methods in bioinformation contributed greatly to bridge genes signatures and cancer characteristics. For example, The Cancer Genome Atlas (TCGA) program and Gene Expression Omnibus (GEO) database both offer abundant resources for cancer investigation. The Least Absolute Shrinkage and Selection Operator (LASSO) regression can both adjust the complexity and execute variable selection, thereby improving the prediction precision and interpretability of the regression model [19]. Moreover, compared with bio-enrichment methods based only on differentially expressed genes (DEGs), Gene Set Enrichment Analysis (GSEA) could take into account those genes with subtle expression changes but significant biological significance, therefore, it is more comprehensive and precise [20]. Herein, we tried to build a gene-based survival score (SS) for LUAD via systematic transcriptome analysis, and this SS exhibited favorable predictive efficacy in multiple datasets.

## Methods

### Transcriptomic and clinical information

LUAD datasets containing gene expression profiling and clinical information were obtained from TCGA program (RNA-sequencing) and GEO database (gene microarray). We applied different datasets to different analysis based on the data characteristic and analytic demands, as follows. Four datasets consisting of transcriptome profiling in tumors and normal tissues were applied for filtrating DEGs (GSE32863, 58 tumors and 58 normal tissues; GSE43458, 80 tumors and 30 normal tissues; GSE10072, 58 tumors and 49 normal tissues; TCGA-LUAD, 58 paired tumors and normal tissues) [21–24]. Four datasets containing non-controversial and available records about Overall Survival (OS) and Progression-free Survival (PFS) were applied for survival analysis (TCGA-LUAD, 402 samples; GSE30219, 85 samples; GSE31210, 200 samples; GSE50081, 124 samples) [24–27]. TCGA-LUAD was used as training set while GSE30219, GSE31210 and GSE50081 were applied as validation sets.

Besides, 515 samples of TCGA-LUAD, GSE30219, GSE31210 and GSE50081 were candidates for enrichment analysis. And 253 samples of TCGA-LUAD containing comprehensive clinicopathologic records (age, gender, TNM parameters, clinical stage, OS and PFS) were applied for multivariate analysis.

### Statistical methods

DESeq2 package (RNA-sequencing) and limma package (microarrays) were applied to DEGs (Adjusted *P*-value < 0.05, fold change > 2 or < 0.5) [28, 29]. Z score was used to normalize data (Function: scale). Cox regression model, LASSO regression model, Kaplan-Meier (K-M) curve and log-rank test were used for survival analysis (Packages: survival, survminer and glmnet). Principal component analysis (PCA) was utilized for comprehensive assessment (Function: princomp; Packages: FactoMineR and factoextra). Receiver operating characteristic (ROC) curve analysis was performed to determine optimal cut-off (Packages: pROC). Logistic regression model was applied to find associations between genes and two-category data (TNM parameters and clinical stage were transferred to two-category data) (Function: glm). Pearson correlation analysis was applied for correlation assessment (Packages: ggcorrplot). GSEA was employed for biological investigation [20]. Wilcoxon rank sum test was applied for differential analysis between two groups (Function: wilcox.test). *P* < 0.05 was considered significant. Arithmetic functions were operated in R language [30].

## Results

### Identifying 13 core genes to establish SS for LUAD

**Fig. 1:**
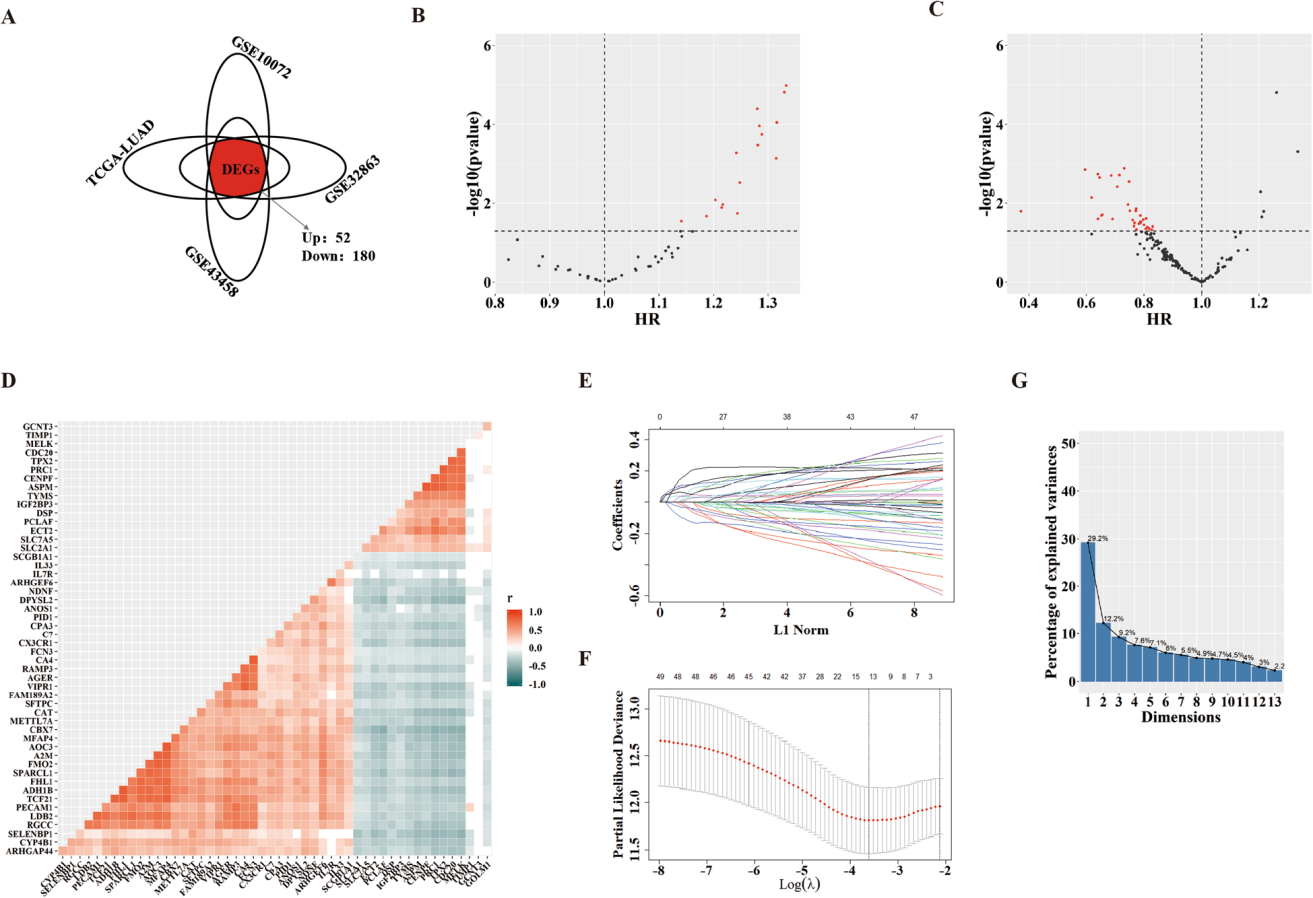
A Survival-related SS for LUAD. **a** DEGs acquired from the intersection of four LUAD datasets (GSE10072, GSE32863, GSE43458 and TCGA-LUAD); **b**, **c** Survival-related genes screened out preliminarily from upregulated DEGs and downregulated DEGs according to OS via univariate Cox regression analysis; **d** The correlation matrix for survival-related genes screened preliminarily; **e**, **f** Core genes identified further considering OS by LASSO regression analysis; **g** Comprehensive assessment of core genes by PCA

Genes closely related to tumor prognosis are likely to play key roles in tumor progression. Valuable candidates are DEGs between tumors and normal tissues. So we obtained the intersection of DEGs from four LUAD transcriptomic datasets (GSE10072, GSE32863, GSE43458 and TCGA-LUAD), and we acquired 52 upregulated DEGs and 180 downregulated DEGs (fold change > 2 or fold change < 0.5, Adjusted *P*-value < 0.05) (Fig. 1a) (Detailed information about acquiring DEGs could be seen in our previous research [31]). Filtrating survival-related genes from these DEGs was executed in TCGA-LUAD dataset, for its largest sample size and most complete clinical records. We conducted univariate Cox regression analysis towards OS for preliminary identification, and we obtained 16 hazardous genes from upregulated DEGs and 35 protective genes from downregulated DEGs (for hazardous genes, which may promote cancer, *p* < 0.05, HR > 1; for protective genes, which may prevent cancer, p < 0.05, HR < 1) (Fig. 1b, c). Since independence among variables is a prerequisite for establishing multi-factor models, we analyzed associations among the screened genes through a correlation matrix. The results showed strong relationship among these genes, so further screening was needed (Fig. 1d). Thereupon, we implemented LASSO analysis and we got 13 core genes: Abnormal Spindle Microtubule Assembly (ASPM), Epithelial Cell Transforming 2 (ECT2), Glucosaminyl (N-Acetyl) Transferase 3, Mucin Type (GCNT3), Golgi Membrane Protein 1 (GOLM1), Insulin Like Growth Factor 2 MRNA Binding Protein 3 (IGF2BP3), Solute Carrier Family 2 Member 1 (SLC2A1), Solute Carrier Family 7 Member 5 (SLC7A5), Tissue Inhibitor Of Metalloproteinases 1 (TIMP1), Thymidylate Synthetase (TYMS), Rac/Cdc42 Guanine Nucleotide Exchange Factor 6 (ARHGEF6), Cytochrome P450 Family 4 Subfamily B Member 1 (CYP4B1), Family With Sequence Similarity 189 Member A2 (FAM189A2), and Secretoglobin Family 1A Member 1 (SCGB1A1) (Fig. 1e, f) (Coefficients of genes in LASSO regression are listed in Supplementary Table 1). Furthermore, we performed PCA for the comprehensive evaluation based on core genes (Fig. 1g). Finally, we chose the first 6 principal components (comps) to establish a SS for LUAD (The cumulative contribution rate is more than 70%):

Score = 0.409981*comp.1 + 0.170820*comp.2 + 0.129785*comp.3 + 0.106358*comp.4 + 0.09938*comp.5 + 0.083671*comp.6.

And coefficients for genes to make up each comp are exhibited in Supplementary Table 2.

### SS exhibits high-risk probability for OS in LUAD

**Fig. 2:**
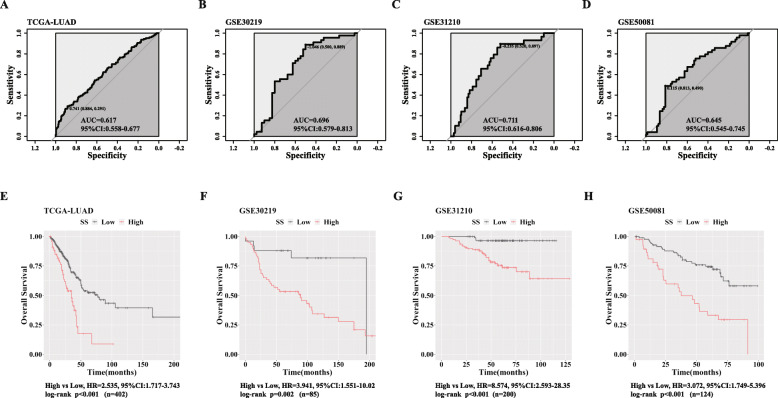
OS rates between LUAD patients with high and low SS. **a-d **ROC curves for determining optimal cut-off for SS upon OS in TCGA-LUAD (**a**), GSE30219 (**b**), GSE31210 (**c**) and GSE50081 (**d**); **e-h **K-M survival curves and HRs considering OS between patients with high and low SS in TCGA-LUAD (**e**), GSE30219 (**f**), GSE31210 (**g**) and GSE50081 (**h**)

Next, we estimated prognostic value of SS in LUAD. We chose OS for prognostic indicator and examined in four independent LUAD datasets (TCGA-LUAD, GSE30219, GSE31210, GSE50081). We divided patients into two groups (High and Low SS) based on optimal cut-off value via ROC curve analysis according to the survival status of OS in each dataset respectively (Fig. 2a-d). We found SS showed obvious high-risk probability for OS and patients with higher SS had shorter OS periods in four datasets (Fig. 2e-h).

### SS possesses high-risk probability for PFS in LUAD

**Fig. 3:**
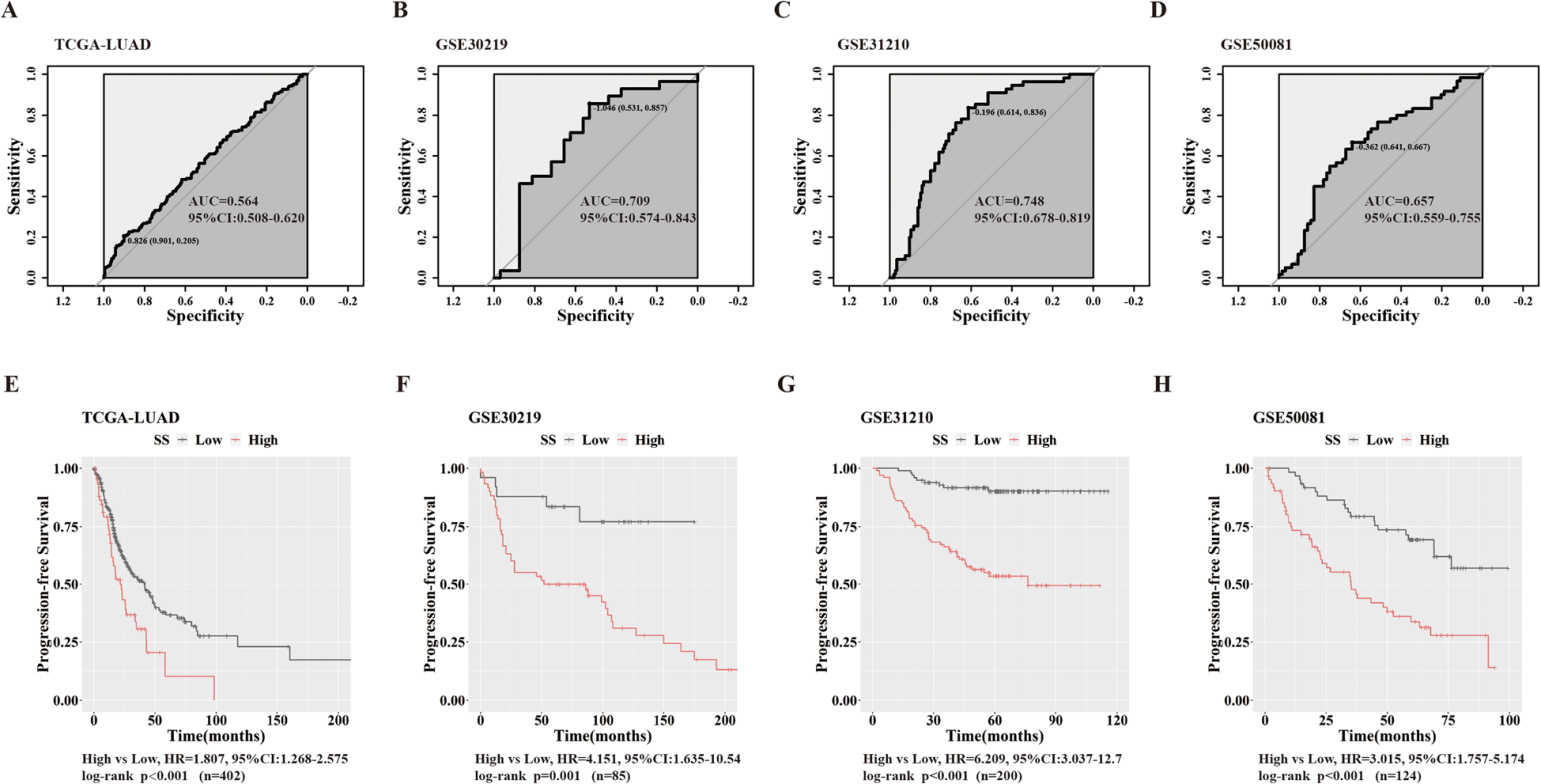
PFS probabilities between LUAD patients with high and low SS. **a-d **ROC curves for finding appropriate cut-off for SS upon PFS in TCGA-LUAD (**a**), GSE30219 (**b**), GSE31210 (**c**) and GSE50081 (**d**); **e**-**h** K-M survival curves and HRs related to PFS between patients with high and low SS in TCGA-LUAD (**e**), GSE30219 (**f**), GSE31210 (**g**) and GSE50081 (**h**)

Then, we estimated predictive value of SS upon PFS in four independent LUAD datasets (TCGA-LUAD, GSE30219, GSE31210, GSE50081). We implemented ROC curve to divided patients into High and Low SS groups based on outcome status of PFS in these datasets respectively (Fig. 3a-d). Analogously, we found higher SS indicated bigger risk probability for PFS in all testing datasets (Fig. 3e-h).

### SS correlates highly with clinicopathological features and functions as a novel independent risk factor for LUAD prognosis

**Fig. 4:**
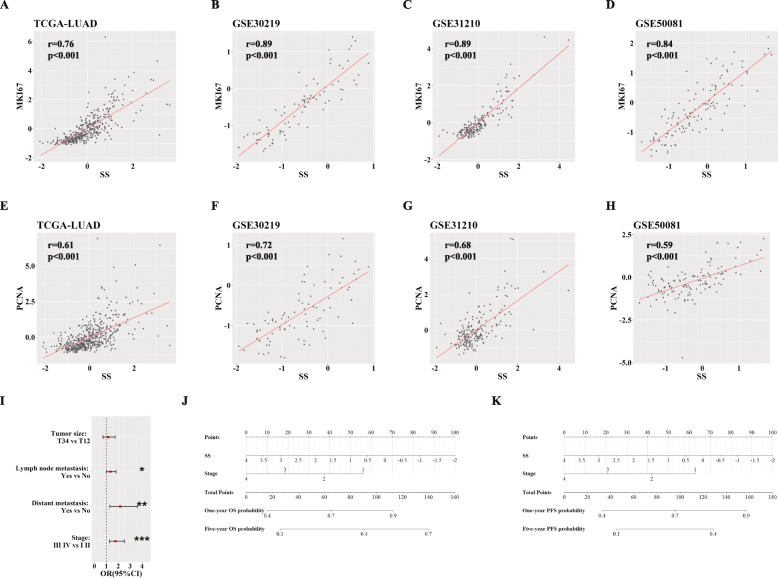
Relationships between SS and clinicopathological features of LUAD. **a-d** Correlations between SS and MKI67 expression in TCGA-LUAD (**a**), GSE30219 (**b**), GSE31210 (**c**) and GSE50081 (**d**); **e**-**h** Correlations between SS and PCNA expression in TCGA-LUAD (**e**), GSE30219 (**f**), GSE31210 (**g**) and GSE50081 (**h**); **i** ORs regarding TNM parameters and clinical stage between patients with high and low SS; **j** The nomograph for predicting one-year and five-year OS probability; **k** The nomograph for predicting one-year and five-year PFS probability

Further, we investigated relationships between SS and clinicopathological parameters of LUAD. Marker of proliferation Ki-67 (MKI67) and proliferating cell nuclear antigen (PCNA) are both canonical biomarkers for clinical oncology [32–35]. We found SS was intensively positively correlated with MKI67 in four LUAD datasets (TCGA-LUAD, GSE30219, GSE31210, GSE50081) (Fig. 4a-d). Analogously, SS possessed strong positive association with PCNA in these datasets (Fig. 4e-h). TNM parameters and tumor clinical stage also play important roles in tumor handling. And we chose TCGA-LUAD dataset with relatively more complete clinical information for following analysis. We found that SS indicated high-risk probability for N (lymph node metastasis), M (distant metastasis) and clinical stage (Fig. 4i). Usually, univariate analysis might cover up the real prognostic function due to confounding factors. So we verified further SS could function as an independent risk predictor for both OS and PFS via multiple Cox regression analysis considering age, gender, TNM parameters and clinical stage in TCGA-LUAD (Table 1, 2). Moreover, we used clinical stage and SS to build concise nomographs predicting OS and PFS probability of LUAD (Fig. 4j, k). And predictive potencies were acceptable (C-indexes: OS, 0.7; PFS, 0.7).

**Table 1:** Cox proportional hazard regression for OS in TCGA-LUAD patients

Parameters	Univariate analysis			Multivariate analysis		
	HR	95%CI	***P*** value	HR	95%CI	***P*** value
**Age (years)**	**0.998**	**0.977–1.019**	**0.833**	**1.013**	**0.991–1.036**	**0.242**
**Gender**	**0.968**	**0.638–1.469**	**0.880**	**0.889**	**0.580–1.364**	**0.591**
**male vs female**						
**Tumor size**	**2.379**	**1.360–4.162**	**0.002**	**2.017**	**1.073–3.790**	**0.029**
**T34 vs T12**						
**Lymph node metastasis**	**2.187**	**1.445–3.310**	**< 0.001**	**1.813**	**1.091–3.014**	**0.022**
**Yes vs No**						
**Distant metastasis**	**1.785**	**0.862–3.699**	**0.119**	**1.204**	**0.482–3.010**	**0.692**
**Yes vs No**						
**Stage**	**2.387**	**1.544–3.691**	**< 0.001**	**1.185**	**0.622–2.259**	**0.605**
**III IV vs I II**						
**Score**	**1.580**	**1.303–1.916**	**< 0.001**	**1.610**	**1.288–2.012**	**< 0.001**

**Table 2:** Cox proportional hazard regression for PFS in TCGA-LUAD patients

Parameters	Univariate analysis			Multivariate analysis		
	HR	95%CI	***P*** value	HR	95%CI	***P*** value
**Age (years)**	**1.002**	**0.985–1.020**	**0.794**	**1.011**	**0.993–1.029**	**0.227**
**Gender**	**0.940**	**0.662–1.335**	**0.731**	**0.906**	**0.632–1.301**	**0.594**
**male vs female**						
**Tumor size**	**2.227**	**1.364–3.638**	**0.001**	**2.041**	**1.160–3.592**	**0.013**
**T34 vs T12**						
**Lymph node metastasis**	**1.606**	**1.131–2.280**	**0.008**	**1.360**	**0.882–2.096**	**0.164**
**Yes vs No**						
**Distant metastasis**	**1.707**	**0.864–3.374**	**0.124**	**1.283**	**0.544–3.027**	**0.569**
**Yes vs No**						
**Stage**	**1.893**	**1.293–2.770**	**0.001**	**1.067**	**0.597–1.907**	**0.829**
**III IV vs I II**						
**Score**	**1.459**	**1.208–1.761**	**< 0.001**	**1.449**	**1.175–1.788**	**< 0.001**

### Exploring molecular characteristics underlying SS in LUAD

**Fig. 5:**
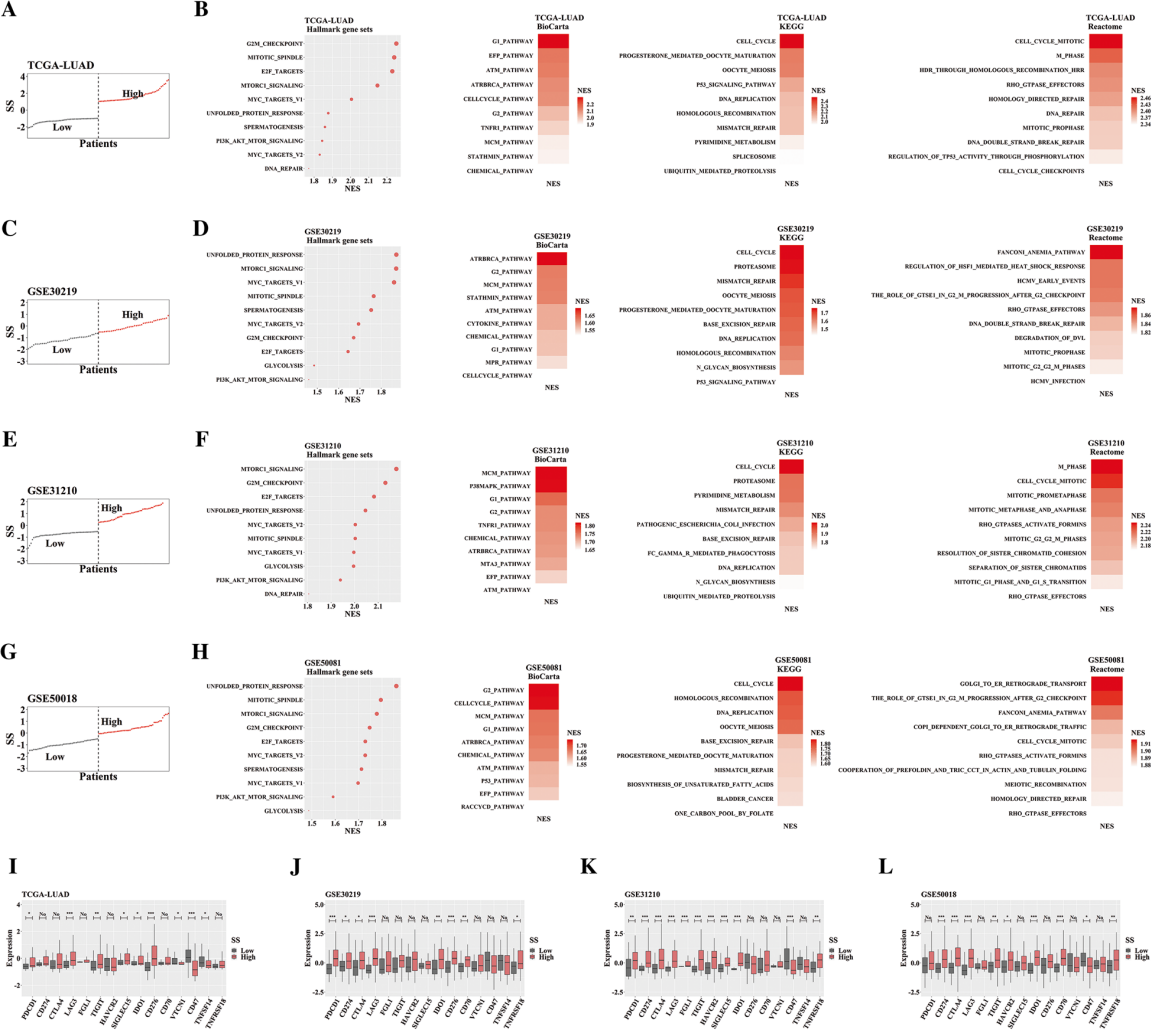
Molecular characteristics underlying SS in LUAD. **a**, **c**, **e**, **g** Dividing LUAD patients into high and low group based on SS in TCGA-LUAD (**a**), GSE30219 (**c**), GSE31210 (**e**) and GSE50081 (**g**); **b**, **d**, **f**, **h** Gene sets enriched in high SS group from several collections of the MSigDB (Only top ten significant gene sets were presented) in TCGA-LUAD (**b**), GSE30219 (**d**), GSE31210 (**f**) and GSE50081 (**h**); **i**-**l** Immune-check genes expression between patients with high and low SS in TCGA-LUAD (**i**), GSE30219 (**j**), GSE31210 (**k**) and GSE50081 (**l**)

We tried to uncover molecular mechanisms underlying clinical role of SS in LUAD. We first ranked the patients (515 samples in TCGA-LUAD) in order of SS. The top 50 patients were divided into High SS group, and last 50 patients were Low SS group (Fig. 5a). Then we performed GSEA to investigate molecular features of SS based on transcription profiling. We found high SS showed enhanced cell cycle in several gene sets (Fig. 5b). Further, we validated it in other three datasets (GSE30219, top 40 vs last 40; GSE31210, top 50 vs last 50; GSE50081, top 50 vs last 50), and found similar results (Fig. 5c-h). Last, we explored expression profile of immune checkpoints under SS in above four LUAD datasets, and found most of immune checkpoints possessed increased expression in high SS group (Fig. 5i-l).

## Discussion

LUAD possesses strong heterogeneities in both tumor biology and clinical characteristics [36]. Therefore, it is urgently needed to precisely assess LUAD prognosis for applying appropriate intervention as well as avoiding overtreatment. Deregulation of gene expression during malignant transformation and progression offers theoretical basis to interpret carcinogenesis via gene signatures, while the tremendous advance in onco-genomics provides prominently practical convenience [6, 14, 16]. Here, we established a gene-based survival assessment named SS, which exhibited good accuracy and stability in multiple datasets.

Compared with former gene-based prognostic predictions [37–40], SS owns three specialties or innovations: 1. SS has favorable stability, or adaptability in clinical application. Our initial candidates are common DEGs between tumors and normal tissues in four independent LUAD datasets. After screening by Cox model and LASSO regression, we established a 13 gene-based SS in TCGA-LUAD, and validated its efficiency in other three LUAD datasets. 2. SS was multifunction for clinical usage. First, SS could assess both OS and PFS. OS is a golden standard of prognosis evaluation but takes a long time to collect, while PFS is a relatively convenient indicator for clinical intervention. Besides, SS was positively related to malignant biomarkers and tumor stage, and could function as an independent risk factor for prognosis. 3. Biological significance of SS was verified in multiple datasets. In four LUAD datasets, higher SS all indicated enhanced cell proliferation, which confirmed the prime trait of abnormal proliferation in carcinogenesis. Moreover, LUAD with higher SS exhibited increased expression of immune checkpoint genes, which underlined the prominent role of immune escape in malignant progression of LUAD.

These 13 core genes building SS are involved in a diversity of biological processes like cell proliferation, nutrition transportation and material metabolism. Most of these genes have been reported to play critical roles in lung carcinogenesis, however, some lack detailed research. For example, ASPM and ECT2 both are proliferation-related genes and participate in DNA synthesis and cytokinesis [41–43]. Studies have shown that LUAD had high expression of ASPM and ECT2, which both indicated poor prognosis, and ECT2 could facilitate lung tumorigenesis [43–49]. Lung cancer also has high expression profile of GCNT3 (a gene regulating mucin synthesis), GOLM1(a gene coding for a Golgi transmembrane protein) and IGF2BP3 (a gene coding for a RNA binding protein), which are all positively correlated with malignant progression of lung cancer [50–53]. Nutrient transporter SLC2A1 and SLC7A5 both exhibit cancer-promoting potential in lung cancer [54–56]. TIMP1 was originally thought to be a tumor suppressor gene, since it could intensely inhibit matrix metalloproteinases (MMPs), canonical oncoproteins [57, 58]. However, recent studies have shown that TIMP1 was highly expressed in LUAD, functioned as an independent prognostic risk factor and could facilitate malignant progression through non-MMPs pathways [58–60]. TYMS, a nucleotide synthetase, is commonly used as an indicator of chemotherapy sensitivity, that is its high expression in lung cancer often indicates insensitive for pemetrexed [61]. Also, for patients under platinum-based adjuvant treatment after surgical resection, high TYMS expression often indicates poorer prognosis [62]. SCGB1A1, coding for secretory globulins, has a protective role against smoking-induced lung tumorigenesis [63]. However, for CYP4B1, ARHGEF6 and FAM189A2, their function in lung cancer are rarely studied.

Of course, there are some flaws in our research. First, the present transcriptomic data mainly covered protein-coding genes, but increasing studies have uncovered that non-coding RNAs like microRNAs (miRNAs), long non-coding RNAs (lncRNAs) and circulating RNAs (cirRNAs) present powerful biological functions, especially in cancer research [64]. Second, the transcriptomic data for analysis came from tissues, while estimation based on liquid detection would be less invasive and more executable [65]. Improvement will be made in our future work.

## Conclusions

In brief, we built a gene-based survival SS for LUAD and proved its wide applicability for clinical predictions, which will assist in handling LUAD effectively.

## Supplementary information

**Additional file 1: Supplementary Table 1**, Coefficients for genes under LASSO regression.

**Additional file 2: Supplementary Table 2**, Coefficients for genes to make up each comp.

## Data Availability

Original data can be found in https://www.ncbi.nlm.nih.gov/geo/ and https://portal.gdc.cancer.gov/.

## Abbreviations

LUAD: Lung adenocarcinoma
SS: Survival score
EGFR: Epidermal growth factor receptor
TCGA: The Cancer Genome Atlas
GEO: Gene Expression Omnibus
LASSO: Least Absolute Shrinkage and Selection Operator
DEGs: Differentially expressed genes
GSEA: Gene Set Enrichment Analysis
OS: Overall Survival
PFS: Progression-free Survival
K-M: Kaplan-Meier
PCA: Principal component analysis
ROC: Receiver operating characteristic
ASPM: Abnormal Spindle Microtubule Assembly
ECT2: Epithelial Cell Transforming 2
GCNT3: Glucosaminyl (N-acetyl) Transferase 3, Mucin Type
GOLM1: Golgi Membrane Protein 1
IGF2BP3: Insulin Like Growth Factor 2 MRNA Binding Protein 3
SLC2A1: Solute Carrier Family 2 Member 1
SLC7A5: Solute Carrier Family 7 Member 5
TIMP1: Tissue Inhibitor Of Metalloproteinases 1
TYMS: Thymidylate Synthetase
ARHGEF6: Rac/Cdc42 Guanine Nucleotide Exchange Factor 6
CYP4B1: Cytochrome P450 Family 4 Subfamily B Member 1
FAM189A2: Family With Sequence Similarity 189 Member A2
SCGB1A1: Secretoglobin Family 1A Member 1
comps: Principal components
MKI67: Marker of proliferation Ki-67
PCNA: Proliferating cell nuclear antigen
miRNAs: MicroRNAs
lncRNAs: Long non-coding RNAs
cirRNAs: Circulating RNAs
AUC: Area under the curve
CI: Confidence interval
MSigDB: Molecular Signatures Database
NES: Normalized Enrichment Score
