# Supplementary material for: A gene-based survival score for lung adenocarcinoma by multiple transcriptional datasets analysis
Source: BMC Cancer. 2020 Oct 31;20:1046. doi: 10.1186/s12885-020-07473-1 (PMC7603718; doi:10.1186/s12885-020-07473-1)
Supplement: Supplementary file 1 — Additional file 1: Supplementary Table 1, Coefficients for genes under LASSO regression. [file 12885_2020_7473_MOESM1_ESM.docx]

| **Gene** | **coef** |
| --- | --- |
| **ASPM** | **0.0566** |
| **ECT2** | **0.1083** |
| **GCNT3** | **0.0551** |
| **GOLM1** | **0.0425** |
| **IGF2BP3** | **0.1024** |
| **SLC2A1** | **0.0349** |
| **SLC7A5** | **0.0147** |
| **TIMP1** | **0.1797** |
| **TYMS** | **0.0274** |
| **ARHGEF6** | **-0.1141** |
| **CYP4B1** | **-0.0369** |
| **FAM189A2** | **-0.0243** |
| **SCGB1A1** | **-0.036** |
